# Pilot-scale testing of natural gas pipeline monitoring based on phase-OTDR and enhanced scatter optical fiber cable

**DOI:** 10.1038/s41598-023-41338-4

**Published:** 2023-08-28

**Authors:** Nageswara Lalam, Paul Westbrook, Khurram Naeem, Ping Lu, Paul Ohodnicki, Nathan Diemler, Michael P. Buric, Ruishu Wright

**Affiliations:** 1 National Energy Technology Laboratory, 626 Cochran Mill Road, Pittsburgh, PA 15236 USA; 2 NETL Research Support Contractor, 626 Cochran Mill Road, Pittsburgh, PA 15236 USA; 3 OFS Labs, 19 School House Road, Somerset, NJ 08873 USA; 4 Mechanical Engineering and Materials Science, University of Pittsburgh, Pittsburgh, PA 15261 USA; 5 OFS Fitel, LLC, 55 Darling Drive, Avon, CT 06001 USA; 6 National Energy Technology Laboratory, 3610 Collins Ferry Road, Morgantown, WV 26505 USA

**Keywords:** Optical sensors, Electrical and electronic engineering

## Abstract

In this paper, we present the results of lab and pilot-scale testing of a continuously enhanced backscattering, or Rayleigh enhanced fiber cable that can improve distributed acoustic sensing performance. In addition, the Rayleigh-enhanced fiber is embedded within a tight buffered cable configuration to withstand and be compatible for field applications. The sensing fiber cable exhibits a Rayleigh enhancement of 13 dB compared to standard silica single-mode fiber while maintaining low attenuation of ≤ 0.4 dB/km. We built a phase-sensitive optical time domain reflectometry system to interrogate the enhanced backscattering fiber cable both in lab and pilot-scale tests. In the laboratory experiment, we analyzed the vibration performance of the enhanced backscattering fiber cable and compared it with the standard single-mode telecom fiber. Afterward, we field validated for natural gas pipeline vibration monitoring using a 4-inch diameter steel pipeline operating at a fixed pressure level of 1000 psi, and a flow rate of 5, 10, 15, and 20 ft/s. The feasibility of gas pipeline monitoring with the proposed enhanced backscattering fiber cable shows a substantial increase in vibration sensing performance. The pilot-scale testing results demonstrated in this paper enable pipeline operators to perform accurate flow monitoring, leak detection, third-party intrusion detection, and **c**ontinuous pipeline ground movement.

## Introduction

Oil and gas pipeline integrity monitoring is an emerging application to fulfill the rising need for safety, productivity, and efficiency in operations^1^. As the world’s pipeline infrastructure grows and ages, regulatory requirements on pipeline safety and reliable operation are increasing as well. Faults in these pipeline systems result in service outages and huge economic losses; furthermore, the leaks cause environmental pollution or even disastrous accidents, particularly when the leaks are not identified in time^2^. In natural gas pipeline infrastructure, the primary fuel constituent is methane, which is a potent greenhouse gas. According to a Rhodium Group report, 2015^3^, global natural gas emissions of about 3.5 trillion cubic feet escape into the atmosphere in a year, which is a significant source of global greenhouse gas emissions. Whereas, nearby excavation damage and aging pipelines remain major causes of pipeline leaks. According to Pipeline and Hazardous Materials Safety Administration (PHMSA) in the US, since 2005, a total of 1052 excavation damage-related incidents occurred in the U.S., resulting in 183 fatalities, and ≈ 1.5 billion in economic damage. Among these incidents, 85% were caused by third-party intrusions^4^. Therefore, continuous and timely monitoring of the entire pipeline infrastructure is critical to ensure high standards of safety and reliability and also to meet federal/state regulatory standards requirement. Therefore, real-time, long-distance pipeline monitoring remains the biggest challenge that needs to be addressed. Some common practices of pipeline leak monitoring systems include; acoustic emission systems, smart pigging, pressure point analysis, patrolling (walking, driving, or with drones), software-based dynamic modeling, infrared spectroscopy, gas/vapor sampling, and radar/camera systems amongst others^5^. Continuous real-time monitoring is done using ground-based systems and is typically confined to high-risk areas where there is a high concentration of components or high population density. However, these methods are labor-intensive and costly procedures for monitoring large pipeline networks, and also typically require a person to travel to a site to perform pipeline monitoring.

Due to the difficulty of monitoring kilometers of pipelines with conventional technology, distributed sensing techniques are preferred. Fiber optic sensor technology is proving to be both field-ready and economically viable for pipeline integrity monitoring to improve overall safety. Typically, distributed fiber optic sensor systems have gained attention in the pipeline industry due to their real-time, remote measurement capability, high sensitivity, and ability to provide continuous monitoring along longer pipeline lengths^6^. Among various distributed fiber sensor systems, a phase-sensitive optical time domain reflectometer (Φ-OTDR) is the most novel technique for real-time acoustic vibration measurement with high sensitivity and spatially resolved sensing^7^. However, when using conventional fibers the sensing performance is severely degraded due to low backscattered intensity level, thus limited sensitivity and performance for pipeline monitoring applications^8^. Moreover, Φ-OTDR sensing performance is severely degraded by interference and polarization fading. On the other hand, increasing the injected pump power for a high backscattering signal can improve the vibration sensing performance, but the maximum pump power is limited by the fiber non-linear thresholds^9^. Therefore, to achieve better sensing performance, increasing the fiber backscattering signal over the longer fiber lengths is crucial. In the literature, several works have been proposed to enhance the backscattering signal, such as weak fiber Bragg grating (FBG) arrays^10,11^, ultra-short and random FBGs^12,13^, and localized reflectors^14,15^. In these methods, the number of reflectors or FBGs are limited to less than a hundred per fiber which restricts longer distance pipeline monitoring applications. In addition, these bare fibers are not appropriate for actual field installation due to fragile nature, and vulnerability to damage. To overcome these limitations, we proposed two approaches: (1) continuously enhanced backscattering with wideband reflections to improve vibration sensing performance, and (2) packaging processed enhanced fiber in a tight buffered cable configuration to endure harsh environments and be compatible with field monitoring.

In this paper, we propose and demonstrate a novel method to monitor the gas pipeline through the acoustic vibration measurement based on a Φ-OTDR (also called a distributed acoustic sensor) system with continuously enhanced Rayleigh fiber cable. In addition, a figure-of-merit is discussed with a theory that enables evaluation of the performance of all types of Rayleigh-enhanced fibers. To our knowledge this was the first time, a Rayleigh-enhanced fiber with a tight buffered cable configuration was demonstrated both in the lab scale and at pilot scale, which enables a pathway for an ultra-sensitive pipeline integrity monitoring solutions.

## Operating principles of phase-OTDR and Rayleigh enhanced fiber cable design

**Figure 1:**
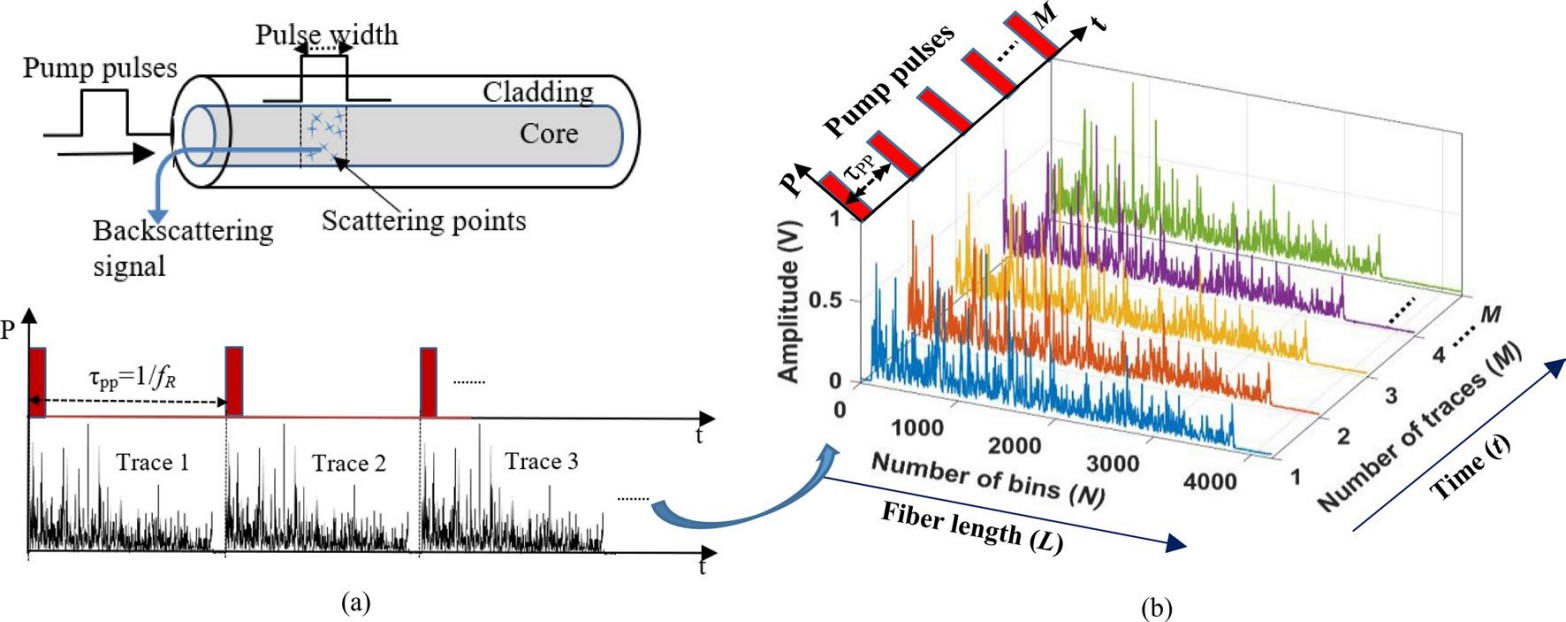
(a) schematic illustration of backscattered Rayleigh phase-OTDR signal, (**b**) illustration of transformed 2-D time-scale matrix of phase-OTDR traces.

In Φ-OTDR, highly coherent light pulses are injected into the sensing fiber, and the backscattered Rayleigh signal is collected at the same end of the sensing fiber, which carries vibration information. The vibration information extracted by a change in intensity and phase of Rayleigh scattering originated from randomly distributed scattering points. The Rayleigh scattering from these scattering centers interferes within a pulse width, where the interference pattern remains constant if the sensing fiber does not experience any external perturbations^16^. When the sensing fiber experiences any localized vibrations, the local interference pattern will change correspondingly. The schematic illustration of Rayleigh backscattering phenomena and reconstructing Φ-OTDR time traces are shown in Fig. 1. A narrow-width pump pulse is launched into the sensing fiber with a pulse repetition frequency, *f*_*R*_ with the pulse period (τ_PP_ = 1/*f*_*R*_), and the number of Rayleigh traces recorded at the receiver. In a Φ-OTDR system, the pulse period should be greater than that of the fiber round trip time to avoid pulses overlapping. Therefore, the pulse period is designed as, τ_PP_ = 2*L*/*v*_g_, where *L* is the sensing fiber length and *v*_g_ is the group velocity of the launched light. The pulse period/pulse repetition frequency determines the vibration sampling or maximum detectable vibration frequency, *f*_max_. According to the Nyquist sampling limit, the maximum detectable vibration frequency is determined as, *f*_max_ = 1/(2(τ_PP_)) ≤ 0.5(*v*_g_/2*L*), whereas, the minimum detectable vibration frequency is determined by the total noise present in the Φ-OTDR system, which comprises thermal and shot noise of the photodetector, and amplified spontaneous emission (ASE). The detected raw data backscattered Rayleigh traces have *M* number of traces (determined by the number of pump pulses), and *N* number of bins (which depends on the number of sampling points), represented by a 2D array matrix [*M, N*], as illustrated in Fig. 1. For distributed acoustic sensing, long-distance weak continuous gratings are required to maintain low attenuation. Here, the Rayleigh-enhanced fiber is fabricated based on a reel-to-reel continuous system using UV exposure and phase mask technology, which enables inscription of localized Rayleigh-enhanced reflectors in kilometers lengths of standard telecommunication single-mode fiber. A detailed fiber fabrication process and production can be found in^17,18^.

**Figure 2:**
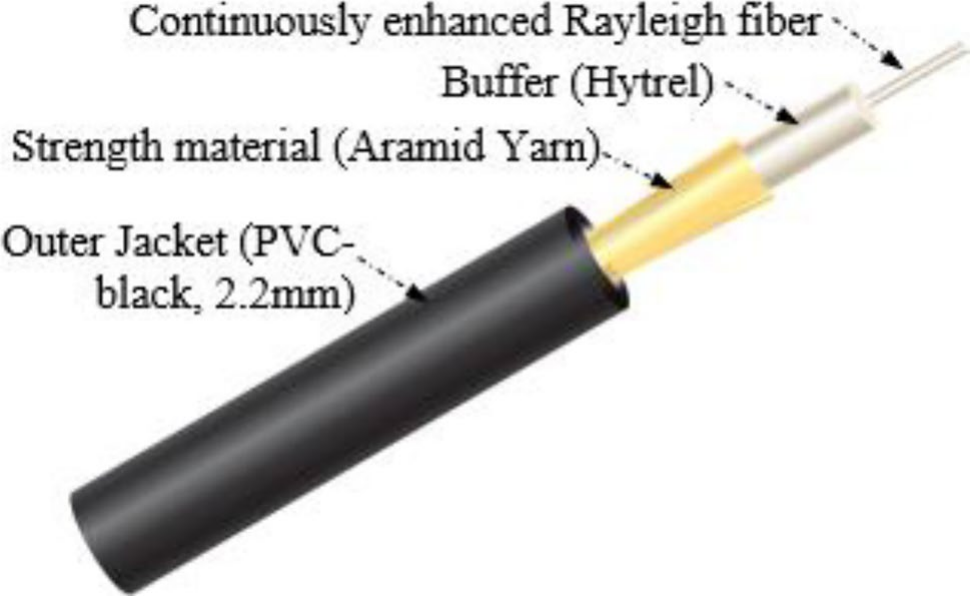
Schematic diagram of wideband Rayleigh enhanced fiber optic cable.

**Figure 3:**
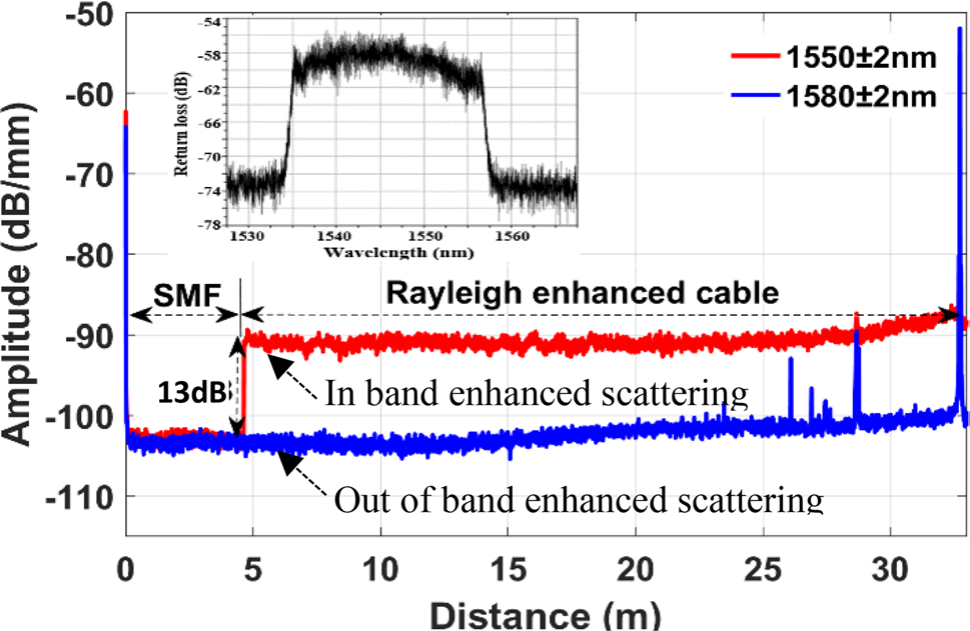
Measured Rayleigh backscattered traces using OFDR. The wavelength tuning range is set at 1550 ± 5 nm (red curve) within the enhanced scattering bandwidth, and 1580 ± 5 nm (blue curve) out of enhanced scattering bandwidth. Inset: Reflection band spectrum measured over 100 m long Rayleigh enhanced fiber cable.

The bare sensor fibers, including the enhanced scattering fiber, are inherently fragile by nature, and need additional mechanical protection to be well-suited for field applications. They are therefore packaged into a fiber cable. The sensing fiber cable is constructed with a 500 μm buffered Rayleigh-enhanced fiber surrounded by aramid yarn strength members jacketed in a PVC outer jacket. The tight-buffer configuration is optimized to improve acoustic wave coupling from the surrounding medium to the fiber. The schematic diagram of Rayleigh enhanced fiber cable is illustrated in Fig. 2. This Rayleigh-enhanced fiber cable has a wide-band operating wavelength (1538–1554 nm), and low attenuation of ≤ 0.4 dB/km while maintaining an enhanced signal-to-noise ratio of ~ 13 dB over the telecom-grade standard single-mode fiber. In order to measure the Rayleigh backscattered signal from the enhanced scattering fiber cable and compare it with conventional silica single-mode fiber (SMF), we spliced a short length (~ 5 m) of standard silica SMF to the enhanced scattering fiber cable. The measured Rayleigh traces using optical frequency domain reflectometry (OFDR, Luna OBR 4600) are illustrated in the Fig. 3. The red trace was measured when the OFDR wavelength tuning range was set at 1550 ± 2 nm, which is within the enhanced scattering bandwidth, and the blue trace at 1580 ± 2 nm, which is completely out of the enhanced scattering bandwidth. As shown in Fig. 3, we can observe approximately 13 dB of Rayleigh gain enhancement compared to standard SMF. We also measured a reflection band spectrum as shown in Fig. 3 inset, which has a wide bandwidth of approximately 16 nm (from 1538 to 1554 nm). Moreover, we found that the attenuation is as low as 0.4 dB per kilometer, which makes this enhanced Rayleigh fiber cable suitable for long-distance measurement.

### Figure-of-merit for Rayleigh backscattering

Recently, there have been many reports of specialty optical fiber with enhanced Rayleigh backscattering signal more than 10 dB above the Rayleigh backscattered signal in the silica SMFs. Although increasing the Rayleigh backscattering signal can significantly enhance the vibration sensing performance, it may also cause substantial fiber attenuation. If the fiber attenuation rises more than a certain level due to high Rayleigh backscattering then the sensing distance is strictly limited (typically few meters), which is not suitable for long-distance applications, (e.g., pipelines). A figure-of-merit (FoM) is an appropriate factor to fairly compare the performance of Rayleigh-enhanced fibers and standard silica SMFs. Therefore, we apply the analysis of a recently discussed FoM for such Rayleigh-enhanced specialty fibers and conventional single-mode fibers to the fibers in our recent study^19^. This FoM offers users the possibility to evaluate the enhanced performance of Rayleigh-enhanced fibers. To clarify the meaning of this FoM, we review its derivation in the context of the single mode fibers we consider in our present work.

Assuming the fiber exhibits only Rayleigh scattering, the backscattered power distribution over the fiber distance, *L* measured at the receiver can be expressed as^20,21^:

1 $$P_{R} \left( L \right) = 0.5 P_{P} W \alpha_{R} \left( L \right)v_{g} S\left( L \right)e^{{\left( { - 2\alpha_{t} L} \right)}}$$

where $P_{P}$ is the input pump power injected into the sensing fiber, $W$ is the pulse width, $\alpha_{R}$ is the Rayleigh scattering coefficient, $v_{g}$ is the group velocity, $\alpha_{t}$ is the total fiber attenuation, and $S$ is the fiber’s backscattered capture fraction, which measures the amount of collected backscattered signal within the numerical aperture of the fiber and depends on the fiber waveguide properties. For a fair proposal of FoM that is suitable for all single mode Rayleigh-enhanced fibers, we separate the waveguide properties and the inherent scattering. Considering the input pump power level (which is limited by the fiber nonlinear threshold level), pulse width at a desired spatial resolution, and fiber attenuation as an optimized value, with no significant impact on the signal-to-noise-ratio at the detector, Eq. (1) can be assumed as $P_{R} \propto \alpha_{R} S$. The capture fraction $S$ for a silica SMF can be expressed as^22^:

2 $$S = \frac{3}{{2n^{2} W_{0}^{2} \left( {\omega /c} \right)^{2} }}$$

where $W_{0}$ is the spot size or mode field diameter, and *n* is the fiber refractive index. When the normalized frequency (*V*-number) for single mode step index fibers $\left[ {V = \frac{2\pi a}{{\lambda_{p} }} \left( {NA} \right), \;where\;NA = \sqrt {n_{1}^{2} , - n_{2}^{2} } } \right]$ is introduced, then the capture fraction $S$ is rewritten as:

3 $$S = \frac{3}{2}\frac{1}{{\left( {W_{0} /a} \right)^{2} V^{2} }}\frac{{\left( {NA} \right)^{2} }}{{n_{1}^{2} }}$$

where *NA* is the numerical aperture, $a$ is the core radius, and $n_{1}$ is the fiber core refractive index. According to Marcuse^19^, for a standard SMF, the normalized spot size $\left( {W_{0} /a} \right)$ dependence on the *V*-number is given by:

4 $$\frac{{W_{0} }}{a} = 0.65 + 1.619 V^{ - 1.5} + 2.879 V^{ - 6}$$

From the above Equation, the capture fraction, $S$ is approximately constant for $1.5 \le V \le 2.4$. Considering *V* = 1.95, which is the average value over a constant *S*, and substituting into Eq. (4), the attained normalized spot size $(W_{0} /a)$ value is 1.29. Replacing this $W_{0} /a$ value into Eq. (3), the capture fraction $S$ for an SMF can be derived as,

5 $$S = 0.235\frac{{\left( {NA} \right)^{2} }}{{n_{1}^{2} }}$$

The approach we have taken for capture fraction calculation can be applied to all step-index SMFs. Therefore, enhancing the backscattering signal can be accomplished either by increasing Rayleigh backscattering over refractive index modifications within the fiber waveguide or by increasing the capture fraction by higher *NA*, as described above.

Based on the above-mentioned understanding of the relationship between capture fraction, *S,* and the waveguide parameters and considering the above Equations, the following unitless FoM applicable to single mode step index fibers can be defined for Rayleigh backscattering:

6 $$FoM = \frac{{P_{R} n_{1}^{2} }}{{ 0.235 \alpha_{t} \left( {NA} \right)^{2} }}$$

where $P_{R}$ is the backscattered Rayleigh power per unit length, and $\alpha_{t}$ is the total fiber attenuation, in which consistent units must be used (see Appendix 1). From Fig. 3, the measured backscattered Rayleigh power value of standard SMF is $P_{R}$ = − 103.3 dB/mm. The fiber attenuation, $\alpha_{t}$ = 0.25 dB/km, *NA* = 0.13, $n_{1}$ = 1.45 (obtained from manufacturer datasheet), the calculated FoM is 0.36 for the SMF. From Fig. 3, the measured value for Rayleigh enhanced fiber is $P_{R}$ = − 90 dB/mm. The measured fiber attenuation is $\alpha_{t}$ = 0.4 dB/km. Accordingly, the FoM is 5.7 for the Rayleigh-enhanced fiber. Detailed calculations can be found in Appendix 1. A 13 dB Rayleigh enhancement over standard SMF leads to a FoM of 5.7, whereas the FoM for SMF is 0.36, which is less than unity as expected due to the presence of excess loss beyond Rayleigh scattering. It is important to emphasize that the higher amount of backscattering outweighs the increased attenuation, leading to a higher FoM.

## Experimental setup and lab-scale demonstration of pipeline monitoring

**Figure 4:**
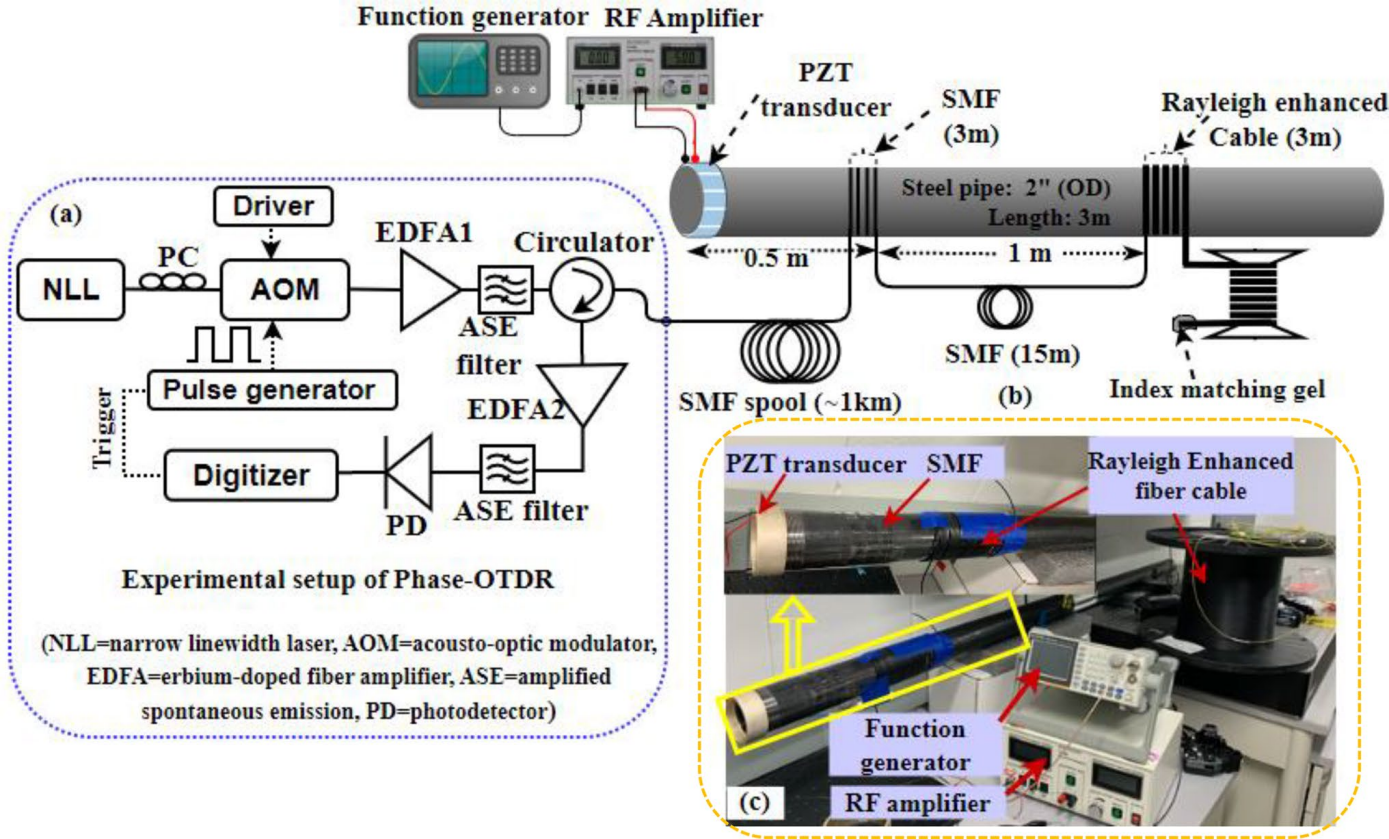
(**a**) Experimental setup of phase-OTDR system, (**b**) Schematic illustration of lab-scale pipe setup installed with SMF, Rayleigh enhanced cable, and PZT excitation source, and (**c**) Picture of a laboratory test setup.

**Figure 5:**
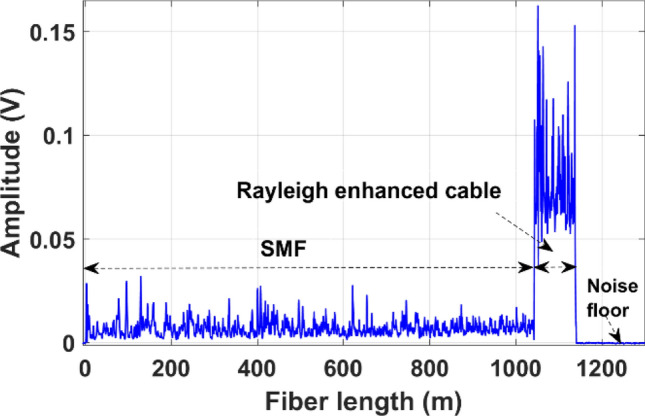
Measured phase-OTDR trace from the sensing fiber.

As illustrated in Fig. 4a, the experimental setup of the direct detection Φ-OTDR system used a highly coherent narrow linewidth laser (NLL, 1 kHz) operating at a wavelength of 1550.12 nm. A polarization controller (PC) was used to control the light polarization and then modulated by an acoustic-optic modulator (AOM) with a 200 MHz frequency shift driven by the external arbitrary waveform generator to generate pump pulses. The extinction ratio of the pulses was about 50 dB. The pulse width is set at 20 ns (which corresponds to 2 m spatial resolution) with a repetition frequency of 80 kHz. Then, the output signal was amplified by an erbium-doped fiber amplifier (EDFA 1, 28 dB gain, and < 5 dB noise figure) to an optimized input power level to the sensing fiber. A narrow band-pass filter (0.8 nm bandwidth) was used to eliminate the amplified spontaneous emission (ASE) noise that originates from the EDFA. Thereafter, the pump pulses are injected into the sensing fiber using a circulator. The Rayleigh backscattered signal is then amplified by another EDFA2 (24 dB gain and < 4.5 dB noise figure), and the unwanted sideband components and ASE noise were eliminated using a narrow band-pass filter. Finally, the received signal is detected by the AC-coupled photodetector (bandwidth: 250 MHz) and digitized at 250 MS/s. Figure 4b provides a schematic illustration of the laboratory test pipe setup. The standard SMF of 3 m and Rayleigh enhanced fiber cable (3 m) were circumferentially wrapped to the 2-inch outer diameter (OD), 10-foot-long steel pipe at one end of the pipe segment, where 15 m of delay fiber was used in between as illustrated in Fig. 4b. The 15 m delay fiber was placed into an acoustically insulated vibration resistance foam box to avoid vibration impact originating from a piezoelectric transducer (PZT). The measured Φ-OTDR trace from the sensing fiber is illustrated in Fig. 5, which consists of ~ 1 km long standard SMF (Corning SMF-28e) spliced to a 100 m long Rayleigh enhanced fiber cable. This trace clearly demonstrates an improved amplitude response in measured Φ-OTDR trace from the enhanced fiber cable.

**Figure 6:**
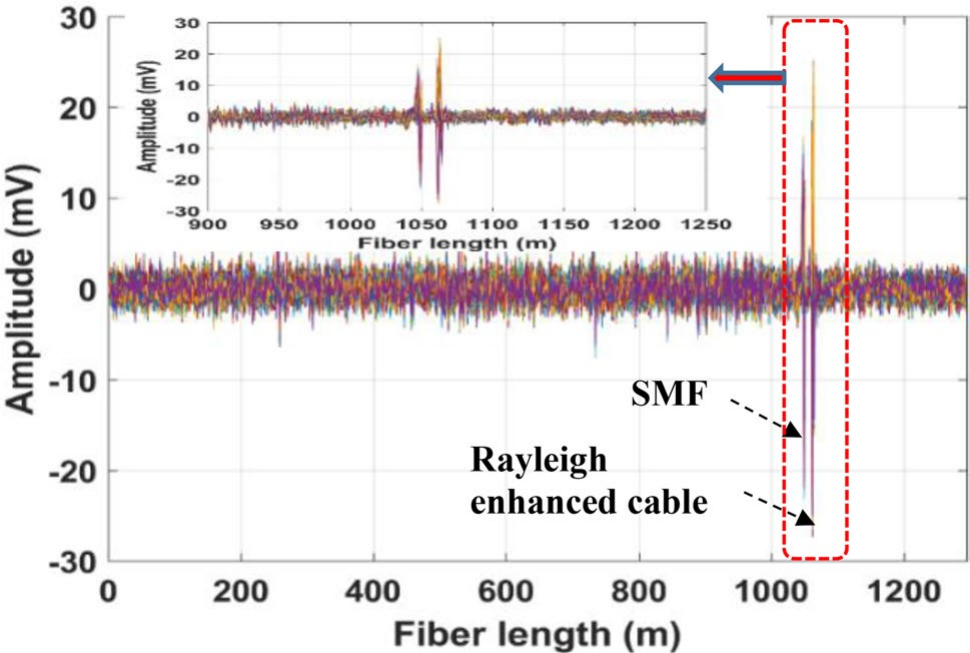
The vibration location after superimposed consecutive trace subtraction. Inset: zoomed trace of vibration location of SMF and Rayleigh enhanced fiber cable.

**Figure 7:**
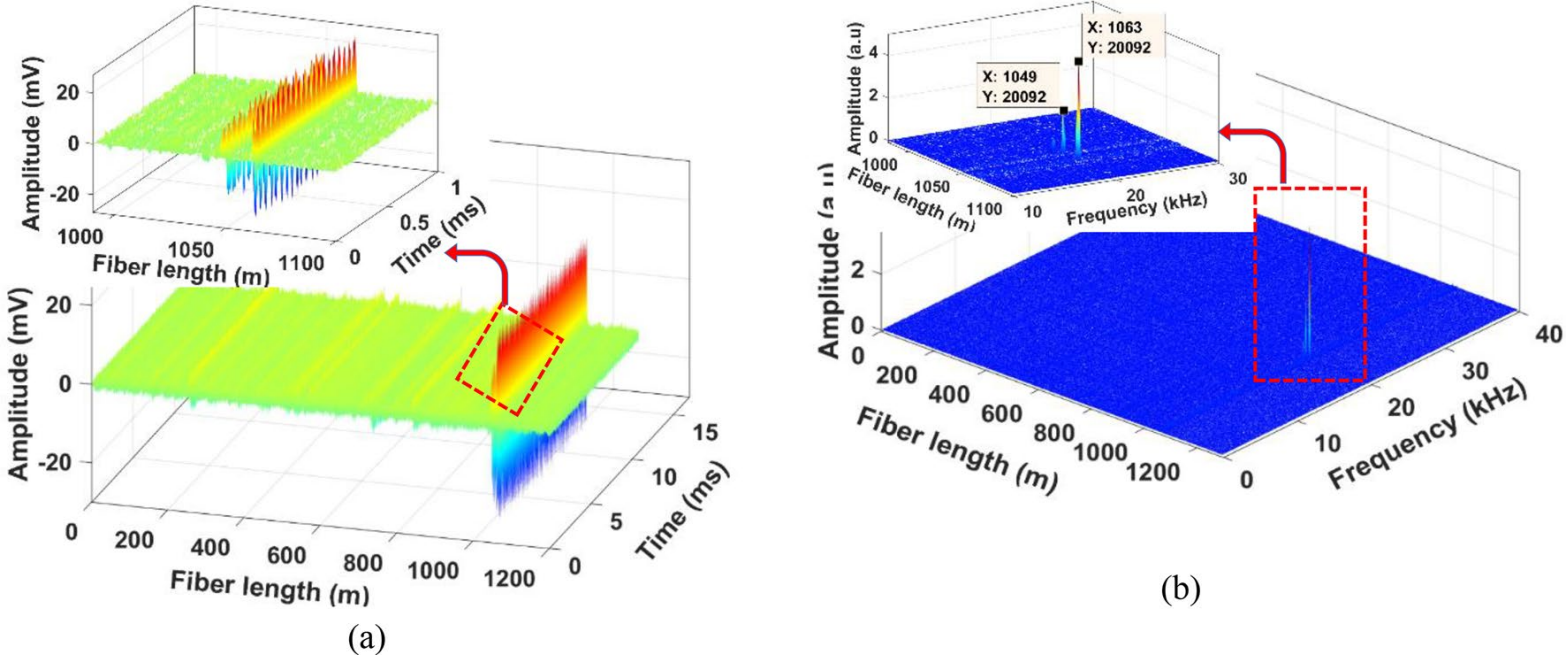
3D spectra at 20 kHz sinusoidal vibration on pipeline (**a**) time-domain (**b**) corresponding frequency domain. Insets: zoomed view of corresponding time/frequency domain plots of SMF and Rayleigh-enhanced fiber cable.

**Figure 8:**
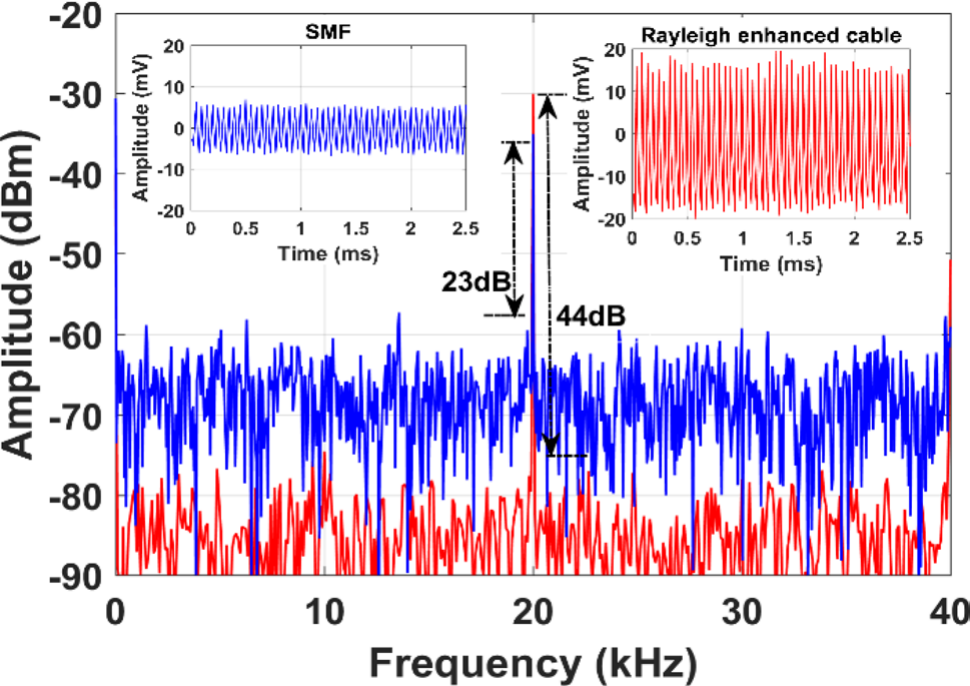
Measured frequency response of standard SMF (blue curve), and Rayleigh enhanced fiber cable (red curve) for an applied vibration of 20 kHz to the steel pipe. (inset: corresponding time-domain traces).

A PZT was attached to one pipe end to generate well controlled acoustic disturbances, which propagate along the pipe length. It should be noted that the PZT cylinder was custom designed for tight-fitting to the 2-inch OD steel pipe to excite acoustic vibrations uniformly around the pipe surface. For comparison, we mounted the same length of 3 m fiber (SMF and enhanced fiber cable) using a strong epoxy adhesive. The locations of the PZT transducer, SMF, and Rayleigh-enhanced fiber cable are illustrated in Fig. 4b. The overall laboratory test setup including PZT attached with steel pipe, and proposed Rayleigh fiber cable is shown in Fig. 4c. Initially, the PZT was driven by a function generator and low-frequency RF amplifier. A sinusoidal excitation frequency at 20 kHz and amplitude at 5V_pp_ is applied to the PZT cylinder, and a set of 2, 200 Φ-OTDR raw traces at no signal averaging was recorded. After subtracting the consecutive traces, the vibration locations of both SMF and enhanced fiber cable mounted on the pipeline can be observed as shown in Fig. 6. The reconstructed time-domain signal, where the axes show the fiber distance, time, and amplitude is illustrated in Fig. 7a. The spatial-frequency 3D spectra were obtained by calculating the fast Fourier transform (FFT) of the reconstructed time-domain signal at each point (also called bins, as shown in Fig. 1b) over the sensing fiber. The resultant frequency domain spectra are shown in Fig. 7b. The two peaks in Fig. 7 correspond to SMF, and enhanced fiber cable mounted on the steel pipe, where the two peak locations are measured at 1049 m and 1064 m, respectively. The gap between these two fiber segments resulted from a 15 m delay fiber used in the test pipe setup. The zoomed plot shown in the Fig. 7 insets clearly illustrates two peaks in both frequency and time-domain 3D spectra and precisely indicate the vibrations applied to the two fiber segments (where PZT is driven by a 20 kHz frequency and 5V_pp_ amplitude). The extracted time domain signal from the SMF (at a distance of 1049 m) and enhanced fiber cable (at a distance of 1064 m) are depicted in Fig. 8 inset. The measured frequency spectra of both standard SMF and Rayleigh-enhanced fiber cable are shown in Fig. 8. A strong frequency peak at 20 kHz is clearly noticeable, which matches the applied sinusoidal vibration frequency of 20 kHz to the steel pipe. The calculated SNR of the measured frequency spectra from SMF is 23 dB, while the SNR of the Rayleigh-enhanced fiber cable is 44 dB. Thus, the SNR improvement at 20 kHz applied frequency is 21 dB compared to standard SMF. These results clearly indicate that the amplitude of the time trace/frequency spectra of the enhanced fiber cable is significantly higher than the standard SMF, and shows a lower noise level with improved SNR.

**Figure 9:**
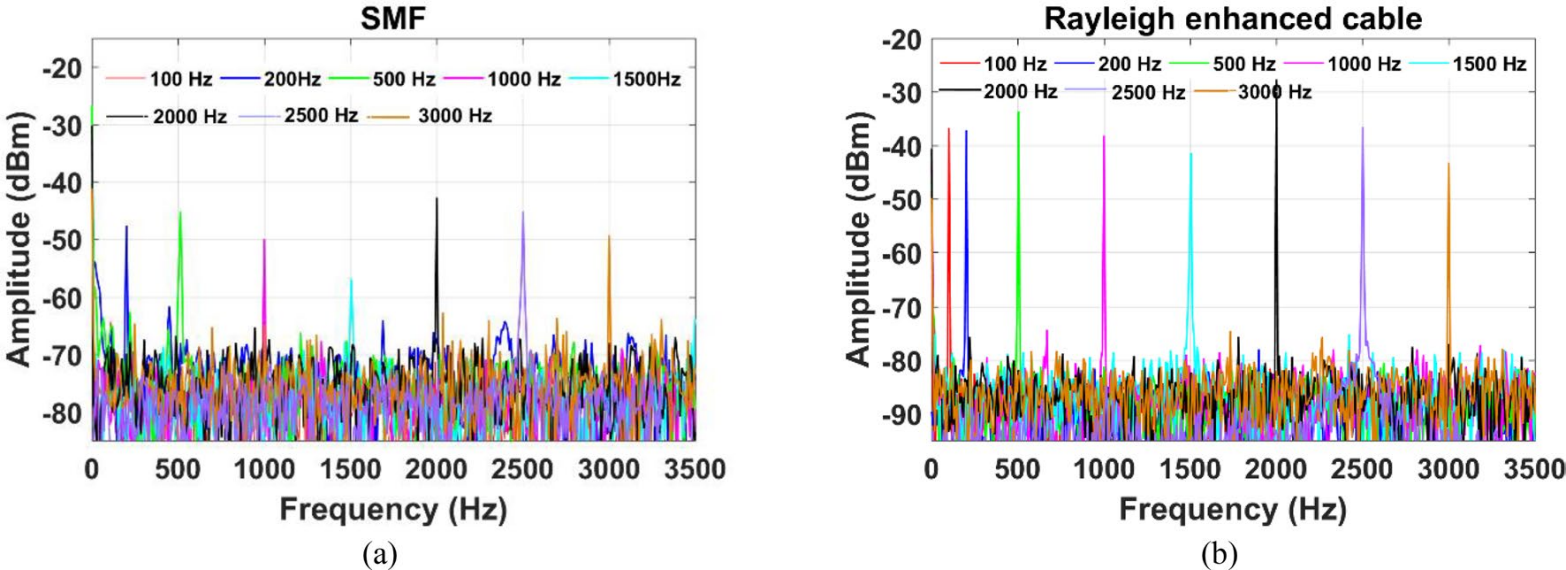
Measured frequency spectral responses of (**a**) standard SMF (blue curve), and (**b**) Rayleigh-enhanced fiber cable at a pipeline excitation frequency from 100 to 3000 Hz.

To further demonstrate the vibration performances, various perturbation frequencies were applied to the steel pipe and the measured frequency spectra are illustrated in Fig. 9. The PZT vibrations were altered from 100 Hz to 3 kHz sinusoidal signal at an amplitude of 5Vpp. At each frequency, the extracted time domain and its frequency spectrum were calculated. It should be noted that other parameters of the experimental system remain unchanged for all frequencies. As shown in Fig. 9b, considerable improvement is evident in detected vibration SNR while lowering the noise floor compared to standard SMF. The acoustic SNR was increased by 29, 18, 17, 16, 20, 19, 11 and 11 dB at the frequency of 100, 200, 500, 1000, 1500, 2000, 2500 and 3000 Hz, respectively. It is evident that the enhanced fiber cable exhibits an improved vibration sensitivity by increasing Rayleigh backscatter while adding little attenuation thereby lowering the noise floor of the sensor to improve the acoustic SNR of the sensing system. Although, the Rayleigh enhanced cable demonstrated here can also be applied for other advanced Φ-OTDR systems^23–25^.

## Pilot-scale demonstration of natural gas pipeline monitoring

**Figure 10:**
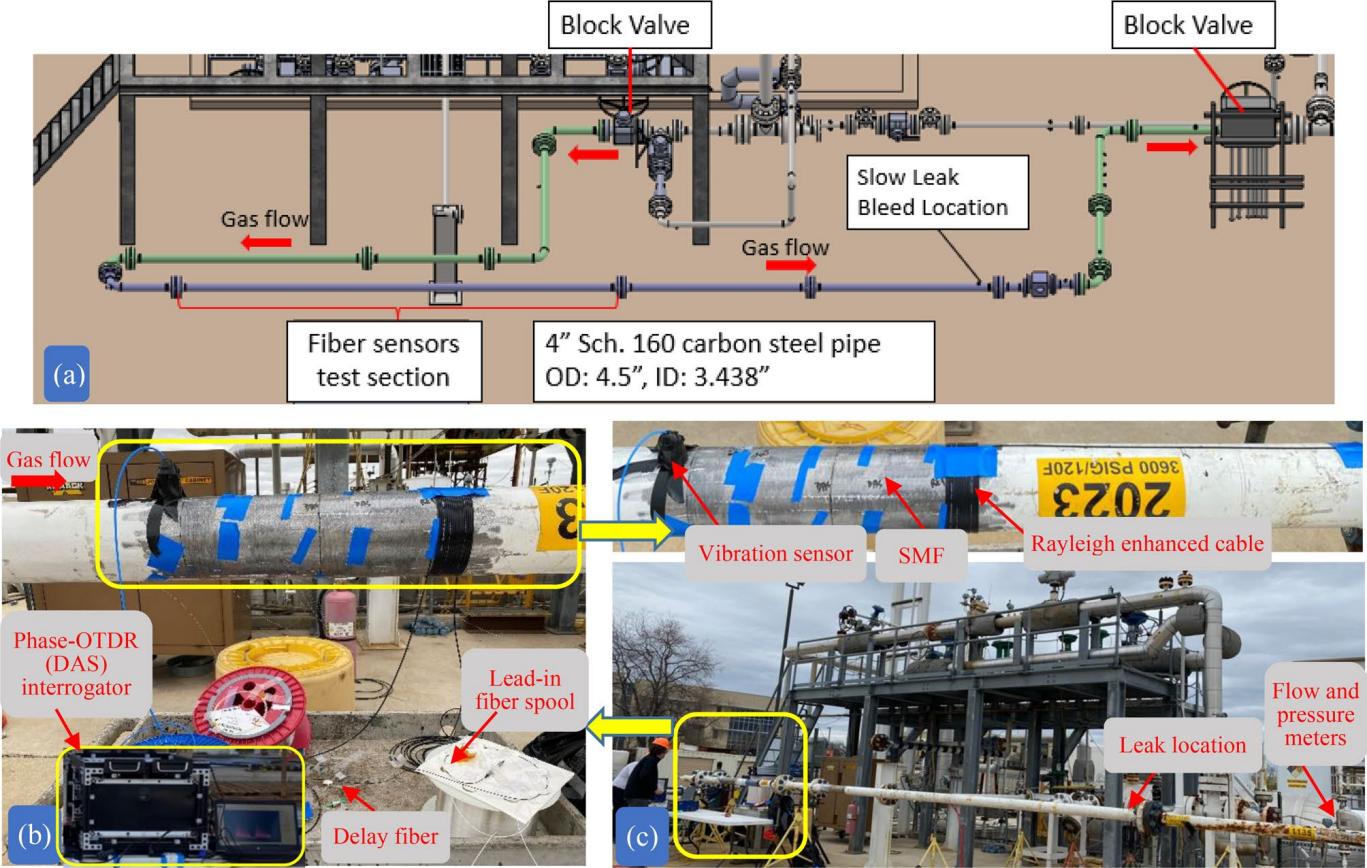
(**a**) Schematic diagram of the high-pressure loop natural gas pipeline system, (**b**) pipeline test section mounted with standard SMF, electrical vibration sensor, and Rayleigh enhanced fiber cable, (**c**) overall high-pressure loop natural gas pipeline test facility at the Metering Research Facility, Southwest Research Institute.

After validating the vibration sensing performance of standard SMF and Rayleigh enhanced fiber cable installed on a 2-inch steel pipe in the laboratory environment, we field demonstrated at a high-pressure loop natural gas pipeline under normal operating conditions. The schematic diagram of the overall closed flow loop, recirculating natural gas flow test section with flow direction, installed fiber sensors and simulated leak location is illustrated in Fig. 10a. The test section pipe consists of 4.5-inch inner diameter (ID), and 245-inch pipe length, which was operated at 1000 psi, and established gas flow rates at 5, 10, 15, and 20 ft/s. The overall picture of the test facility at the Metering Research Facility, Southwest Research Institute (SwRI), San Antonio, Texas, is shown in Fig. 10c. The facility recirculates processed natural gas from the local distribution utility with a simulated pipeline condition.

The sensing fibers were wrapped around the pipe at a dedicated pipe segment using strong adhesive glue. A 6-m long SMF and Rayleigh enhanced fiber cable were installed with a 15-m delay fiber between them. To avoid the gas flow-induced turbulence near the flange, the sensors were installed 1 m away from the flanges. The installed fiber sections on the pipe, delay fiber, and lead-in SMF fiber spool are shown in Fig. 10b. A commercial piezoelectric vibration sensor was also mounted on the pipe to compare with the time traces detected by the Φ-OTDR system. It should be noted that the fiber installation methods can be a helical wrap, circumferential, or along the axis of the pipe (straight). Considering the test pipe section length, we chose circumferential wrap, which also increased the pipe-to-fiber bonding coverage, thus increasing the measurement sensitivity for weak gas flow rates. For permanent monitoring applications for longer pipe lengths, fiber installation along the pipe axis can be considered. It is important to mention that, for buried natural gas pipelines, fiber installation externally to the pipe is challenging and leads to a huge economic burden. Therefore, internal fiber deployment is preferred for retrofitting existing buried pipelines. Recently, we demonstrated a robotic fiber optic deployment tool for internal installation, which can be an effective tool for internal fiber deployment^26^.

**Figure 11:**
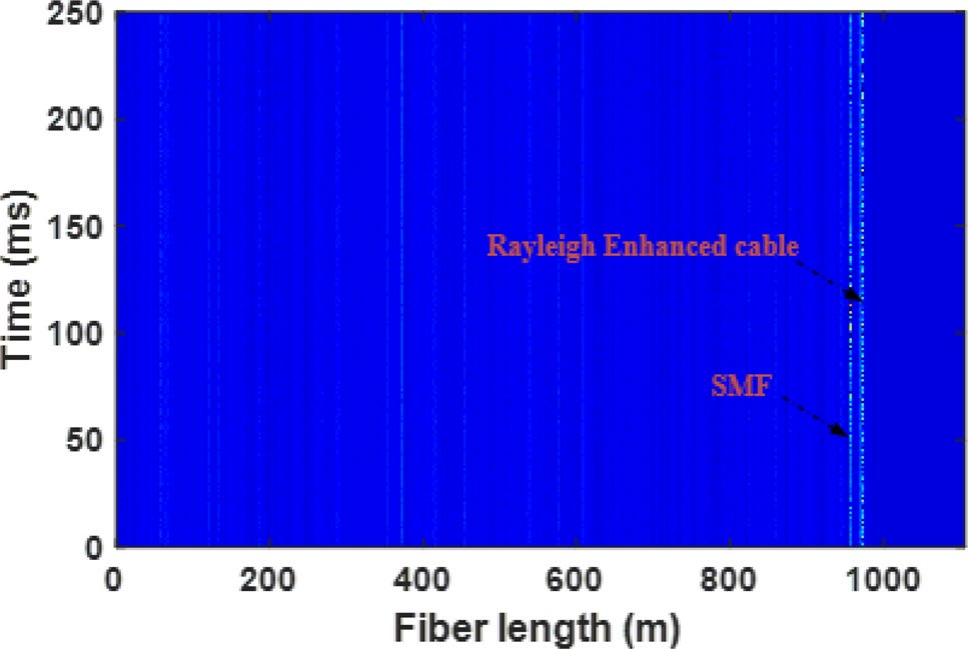
Power spectral density of differential amplitude traces. The gas flow-induced vibrations at fiber installation locations on the pipeline can be observed clearly for both standard SMF and Rayleigh-enhanced fiber cable.

**Figure 12:**
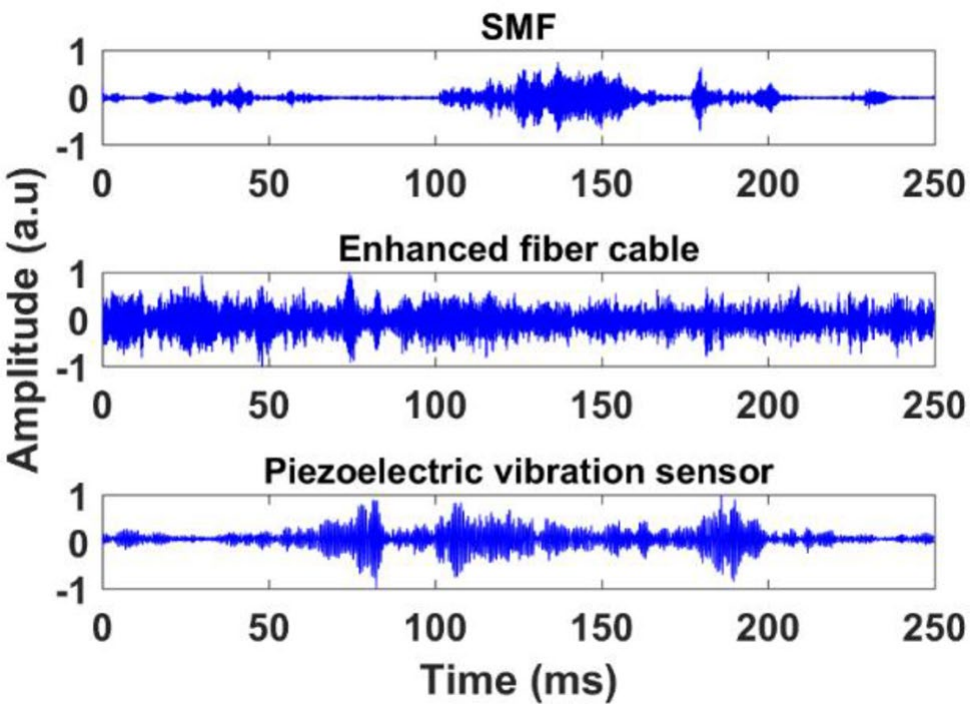
Demodulated time-domain traces of standard SMF, Rayleigh enhanced fiber cable, and piezoelectric vibration sensor at a fixed pressure of 1000 psi and flow rate of 5ft/s.

Initially, the test pipe was purged with dry nitrogen two times to remove atmospheric air and humidity from the test section of the pipe. Next, the test section was filled and purged three times with natural gas to remove residual nitrogen from the line. Then, the test section was filled with natural gas, and flow through the pipe was initiated. The custom built Φ-OTDR interrogator system, shown in the Fig. 10b inset, was placed 2 m away from the test section pipe. The pulse width is set at 20 ns with a pulse repetition frequency of 80 kHz (corresponds to 12.5 µs pulse period), which is slightly greater than the round-trip time of the sensing fiber length. The gas flow-induced vibrations are recorded at a fixed pipe pressure of 1000 psi and flow rate of 5 ft/s. The reconstructed power spectral density plot is illustrated in Fig. 11. It can be seen that, once installed, both installed sensing fibers are clearly noticeable near the end of the total fiber length. Figure 12 illustrates measured time-domain traces in the presence of gas flow of 5 ft/s and 1000 psi. The amplitude level of measured time trace using Rayleigh enhanced fiber cable exhibits a wider range compared to the SMF and piezoelectric vibration sensor, which shows higher vibration sensitivity.

**Figure 13:**
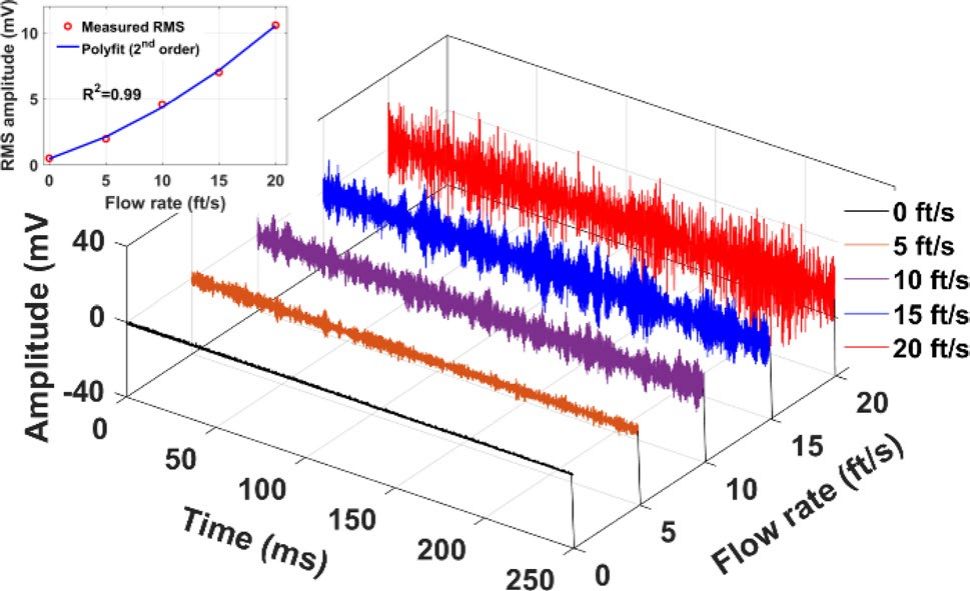
Time-domain traces of Rayleigh enhanced fiber cable at 1000 psi pressure and different gas flow rates of 5, 10, 15, and 20ft/s.

The different responses between the measured time-domain traces that originates from these three sensors are supposed to arise from the various installed locations on the pipeline and experience different vibration modes at distinct times^27,28^. Further, we altered the flow rates at 10, 15, and 20 ft/s at a fixed pressure of 1000 psi, and corresponding vibration traces were measured using Rayleigh enhanced cable, as illustrated in Fig. 13. Each measurement was recorded after flow stabilization at a desired flow rate, and precisely measured using an electronic flow meter and pressure gauge installed on a high-pressure loop pipeline, as shown in Fig. 10c. The flow-induced vibrations that can occur in piping systems due to gas flow generate high kinetic energy that forces the piping system to vibrate and strongly depends on the gas flow rate within the pipeline^29^. The gas flow-induced vibrations in the pipeline originate from the flow generates high kinetic energy that forces the piping system to vibrate^30^. In^31^, the authors investigated the relationship between measured signal noise at variety flow rates is nearly a quadratic trend, which also depends on the pipe material and diameter. From Fig. 13, we can observe that, as the flow rate increases, the measured vibration amplitude fluctuations increase as well. At each flow rate, ten Φ-OTDR measurements were taken, and the average root mean square (RMS) amplitude was calculated and fit to a 2nd-order polynomial curve, which is illustrated in the inset of Fig. 13. A quadratic trend with R^2^ = 0.99 is evident between the measured vibration RMS amplitude and flow rate in the pipeline system.

**Figure 14:**
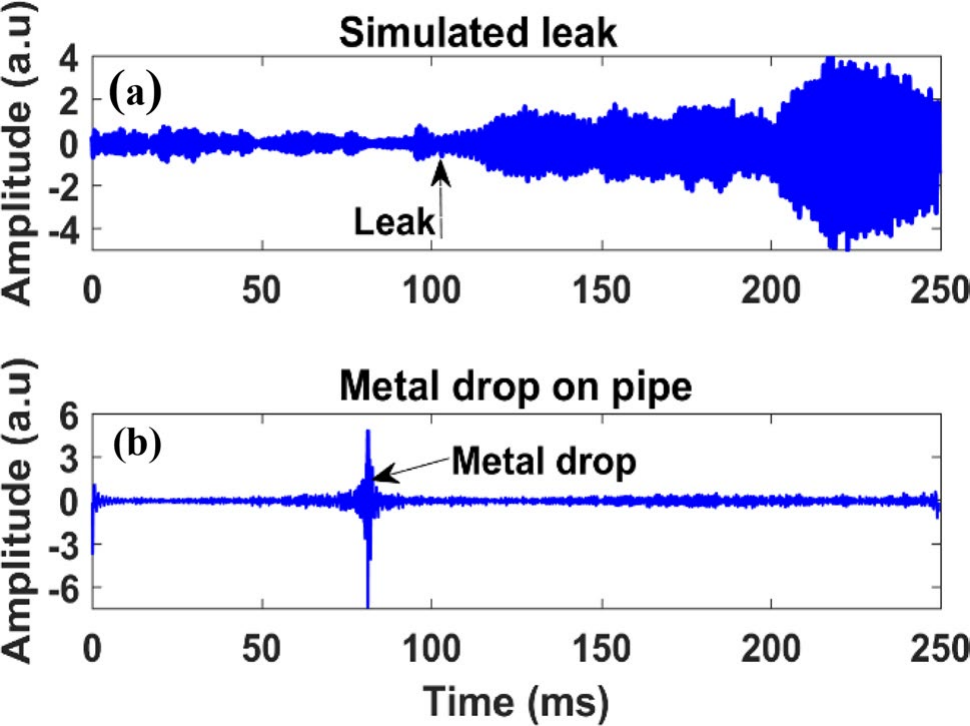
The time domain signals from enhanced fiber during (**a**) control valve leak, and (**b**) metal drop on a pipeline.

Furthermore, pipeline leak detection and third-party intrusion monitoring are highly desirable and help to prevent pipeline damage and catastrophic failures. Therefore, we demonstrated gas leak detection by opening a control valve at one of the flanges (shown in Fig. 10c; the leak location is ∼ 4 m away from the installed fiber sensors), leading to rapid de-pressurization of the pipeline with a distinct vibration signature. Figure 14a shows a measured time-domain trace by the Φ-OTDR system and shows a strong vibration amplitude induced by instant gas leakage. These distinct vibrations with higher amplitude level detected by the sensing fiber are caused by the propagation of the leak-induced vibrations along the pipeline. The simulated leak introduces additional vibrations, and the vibration magnitude depends on the leak size, as well as the leak location from the sensing fiber. After leak detection, the flow rate was stabilized at 5 ft/s and 1000 psi pressure. To demonstrate third-party intruder detection, a small metal bolt was dropped on the pipe, 1 m away from the installed fiber sensors. The metal drop generates extrinsic acoustic signals that propagate along the pipe structure. Figure 14b shows the measure time-domain trace, where the strong amplitude peak originated from the external perturbation to the pipe. Therefore, Fig. 14 clearly demonstrates that the Rayleigh enhanced fiber cable with our custom built Φ-OTDR system is capable of reliable leak and third-party intrusion detection. The pilot-scale demonstration in this work is the fundamental investigation to show the Φ-OTDR system using Rayleigh enhanced fiber cable is highly sensitive and capable of detecting small gas leaks, flow monitoring, and third-party intrusion detection. These sensor signals have potential in pipeline monitoring applications.

## Conclusion

In conclusion, we proposed and field demonstrated a custom built Φ-OTDR system with a Rayleigh-enhanced fiber optic cable for real-time natural gas pipeline monitoring. To be compatible with field monitoring applications, the continuously enhanced backscattering fiber is embedded within a tight buffered cable configuration. The continuously enhanced Rayleigh scattering fiber cable exhibits a 13 dB Rayleigh enhancement compared to conventional silica SMF. We discussed a FoM and detailed analysis to assist the performance evaluation of Rayleigh-enhanced fibers. We then applied the proposed method in laboratory and field tests for pipeline integrity monitoring. Using a direct detection Φ-OTDR system, we experimentally demonstrated a wide-band frequency response up to 20 kHz by adopting a 2-inch OD steel pipe in a lab-scale environment. Finally, the pilot-scale testing results provided the effectiveness of our proposed approach, which enables sensing of flow and leak-induced vibration measurements while protecting pipelines from third-party intrusions.

### Supplementary Information

Supplementary Information.

## Data Availability

The datasets generated and/or analyzed during the current study are not publicly available due to federal lab export control policies but are available from the corresponding author on reasonable request.
