# Supplementary material for: Pilot-scale testing of natural gas pipeline monitoring based on phase-OTDR and enhanced scatter optical fiber cable
Source: Sci Rep. 2023 Aug 28;13:14037. doi: 10.1038/s41598-023-41338-4 (PMC10462723; doi:10.1038/s41598-023-41338-4)
Supplement: Supplementary file 1 — Supplementary Information. [file 41598_2023_41338_MOESM1_ESM.docx]

**Appendix 1**

In this appendix, detailed calculations of FoM for both silica SMF and Rayleigh-enhanced fiber are shown with appropriate unit conversions. Also, the derived equation (6) is explained further for better understanding. We derived FoM for all Rayleigh-enhanced fibers, considering the relation of Rayleigh backscattering ($P_{R}$), total fiber attenuation ${(\alpha}_{t}$), and the backscattered capture fraction (*S*) (which depends on the fiber waveguide design), which can be expressed as follows:

$$FoM=\frac{P_{R}}{\alpha_{t} S}$$

After substituting, capture fraction *S* (Equation (5)) into the above equation,

$$FoM=\frac{P_{R}}{\alpha_{t} 0.235\frac{\left( NA \right)^{2}}{n_{1}^{2}}}\approx\frac{P_{R} n_{1}^{2}}{{0.235 \alpha_{t} \left( NA \right)}^{2}}$$

For multimode fibers, the theoretical values of the Rayleigh backscattering capture fraction were derived by numerous authors. According to M. Nakazawa [1] and E. G. Neumann [2], the step-index and graded-index multimode fibers, the capture fraction *S* is,

$$S_{SI-MMF}=0.38\frac{\left( NA \right)^{2}}{n_{1}^{2}}, and S_{GI-MMF}=0.25\frac{\left( NA \right)^{2}}{n_{1}^{2}}$$

The capture fraction for SMF (equation (5)) is not significantly different from above $S_{SI-MMF}$, $S_{GI-MMF}$ of multimode fibers, given that the *NA* is equal.

**FoM Calculation for SMF:**

Initially, we calculated fiber attenuation using an OTDR device operating at 1550 nm wavelength, and found $\alpha_{t}=$0.28 dB/km=$\left( 0.28\times\frac{log(10)}{10} \right)/1000$=$6.44\times{10}^{-5}/$m. The measured, $P_{R}=$-103.5 dB/mm=-103.5+30 dB/m=${10}^{(-103.5+30)/10}$=$4.46\times{10}^{-8}/$m. The fiber numerical aperture was *NA*=0.13, and core refractive index was $n_{1}$=1.45. Substituting all the above values into the derived FoM equation (6) results in,

$${FoM}_{SMF}=\frac{(4.46\times{10}^{-8})\times{(1.45)}^{2}}{(6.44\times{10}^{-5}) \times0.235\times\left( 0.13 \right)^{2}} =0.36$$

**FoM Calculation for Rayleigh Enhanced Fiber:**

Fiber attenuation was $\alpha_{t}=$0.4 dB/km=$\left( 0.4\times\frac{log(10)}{10} \right)/1000$=$9.21\times{10}^{-5}/$m. The Ryleigh backscattering power was $P_{R}=$-90 dB/mm=-90+30 dB/m=${10}^{(-90+30)/10}$=$1\times{10}^{-8}/$m. A scattering power enhancement of 13 dB was observed compared to the SMF. The fiber numerical aperture was *NA*=0.13, and core refractive index was $n_{1}$=1.45. Substituting all the above values into FoM equation (6) results in,

$${FoM}_{Enhanced fiber}=\frac{(1\times{10}^{-8})\times{(1.45)}^{2}}{(9.21\times{10}^{-5}) \times0.235\times\left( 0.13 \right)^{2}} =5.7$$

**References**

[1] M. Nakazawa, "Rayleigh backscattering theory for single-mode optical fibers," *Journal of the Optical Society of America,* vol. 73, pp. 1175-1180, 1983.

[2] E. G. Neumann, "Analysis of the backscattering method for testing optical fiber cables," *AEU, Electron, and Commun.,* vol. 34, pp. 157-160, 1980.
